# Safety assessment of the *Polypodium feei* root extract: Acute and subchronic studies

**DOI:** 10.1016/j.toxrep.2021.03.013

**Published:** 2021-03-22

**Authors:** Deden Winda Suwandi, Tina Rostinawati, Muchtaridi Muchtaridi, Anas Subarnas

**Affiliations:** a Department of Pharmacology and Clinical Pharmacy, Faculty of Pharmacy, Universitas Padjadjaran, Jatinangor, Sumedang, Indonesia; b Department of Pharmaceutical Biology, Faculty of Pharmacy, Universitas Padjadjaran, Jatinangor, Sumedang, Indonesia; c Department of Pharmaceutical Analysis and Medicinal Chemistry, Faculty of Pharmacy, Universitas Padjadjaran, Jatinangor, Sumedang, Indonesia; d Department of Pharmacy, Faculty of Mathematics and Natural Sciences, Universitas Garut, Garut, Indonesia

**Keywords:** 50% Lethal dose, Macropathology, Mice, Mortality, Proanthocyanidin

## Abstract

This study was performed to assess the safety of the oral acute and subchronic administration of *Polypodium feei* root extract through acute and subchronic studies in mice and rats, respectively. In the acute toxicity treatment, mice were grouped according to the dose (1000, 2000, 4000 and 5000 mg/kg, b.w) and were observed for mortality and toxicity signs for 14 days. In the subchronic treatment, there were six groups of rats (female and male), a control group, three test groups (100, 400, and 800 mg/kg, b.w), and two satellite groups (control satellite and satellite 800 mg/kg groups). The three test groups received the extract orally once daily for 90 days. No animals in the acute and subchronic treatment groups showed mortality and any signs of toxicity, with no significant difference in the body weight and organ index compared to the control. The LD_50_ of the extract was estimated to be higher than 5000 mg/kg, therefore regarded as practically non-toxic. The haematological profiles did not significantly change on exposure to the extract for 90 days, except the platelet count in the female animals which significantly decreased in animals treated with 400 and 800 mg/kg, returning to normal after 28 days of recovery. The 800 mg/kg dose significantly increased the urea concentration and induced lesions in the stomachs of female animals. However, this undesirable effect on the kidney was not strong, as the creatinine concentration remained in the normal limits, and the histopathological observations showed no alteration in the kidney tissues. No significant morphological alterations in organs were observed, only minor lesions in the liver. These results indicate that the *P. feei* root extract is safe for use as herbal medicine and recommended at doses lower than 400 mg/kg.

## Graphical abstract

**Figure:**
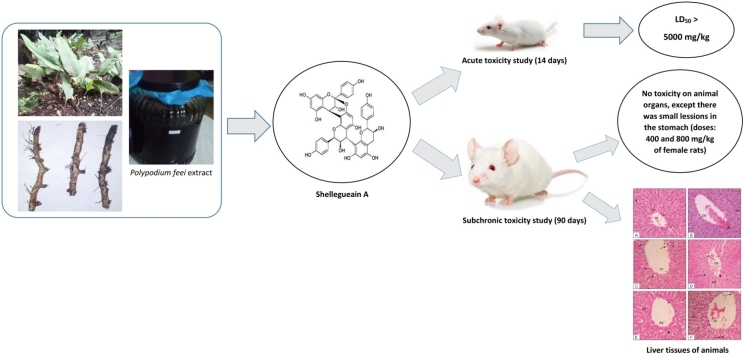

## 1 Introduction

*Polypodium feei* METT is a fern plant of the Polypodium genus in the Polypodiaceae family. This genus widely spreads throughout the world, with the greatest species diversity in the tropics. *P. feei* is an epiphytic or epilithic fern, growing in forests, in open areas, among rocks, on cliffs and roadsides at an altitude of 900−3150 m [1]. In a Java island, it grows on volcanoes near craters because it is resistant to volcanic fumes, and occurs in abundance around the crater of the Tangkuban Perahu mountain in West Java of Indonesia [2].

In the traditional medicine, the roots or rhizomes of *P. feei* have been used as a diuretic and for rheumatism and hypertension, as well as exerting an aphrodisiac effect [2]. Baek et al. (1993) reported a trimeric proanthocyanidin constituent of the *P. feei* roots, named selligueain A, which is rated highly sweet by a taste panel and not toxic for mice in the preliminary acute toxicity test [3]. Furthermore, other substances have been isolated, such as selligueain B (epiafzelechin-(4β→8, 2β→O→7)-epiafzelechin-(4β→8)-3′-deoxydryopteric acid methyl ester), kaempferol-3-O-β-d-glucopyranoside-7-O-α-l-rhamnopyranoside, a bitter-tasting flavonoid glycoside, (-)-4β-carboxymethyl epiafzelechin (3′-deoxydryopteric acid), and (+)-afzelechin-O-β-4′-d-glucopyranoside [4]. Selligueain A and selligueain B are reported to have high antioxidant activity [5].

Previously, we reported that selligueain A of the *P. feei* roots has analgesic and anti-inflammatory activity in mice and rats, respectively, and the proanthocyanidin component inhibited cyclooxygenase activity [2]. Furthermore, we reported the inhibitory effect of the methanol extract of the *P. feei* roots on the occurrence of gastric ulcers induced by stress and HCl/ethanol [6]. These results provided evidence for the human health benefits of the *P. feei* plant, especially for pain management. However, the toxicity of the extract is an important consideration and must be evaluated to guarantee safety. Toxicity is the state of adverse effects due to toxicants, the intensity of which is dependent on the chemical properties of both the toxicants and the cell membrane [7].

This study was intended to examine the toxicity of the *P. feei* root extract using acute and subchronic oral toxicity tests in animal models following the protocols described in the INADFC guidelines [8].

## 2 Materials and methods

### 2.1 Plant materials

The roots of *Polypodium feei* were collected around the crater of the Tangkuban Parahu mountain in the Province of West Java of Indonesia. Collection of the plant roots was conducted in the dry season. This plant was authenticated at the Laboratory of Herbarium Bandungense, School of Life Science and Technology, Bandung Institute of Technology, Indonesia. The roots were washed and air dried out of the direct sunlight. (Herbarium voucher number: 5266/11.CO2.2/PL/2018).

### 2.2 Animals

Swiss Webster female mice weighing 22–27 g and Wistar rats (female and male) weighing 120–150 g were used in the acute and the subchronic toxicity tests, respectively. All animals were kept in the laboratory under controlled conditions at room temperature and were fed with solid foods and water *ad libitum*. The animals were purchased from the Centre of Life Science, School of Life Science and Technology, Bandung Institute of Technology, Indonesia, and acclimatised for one week before the experiment.

### 2.3 Extraction of *P. feei* roots

Dried roots of *P. feei* (4.5 kg) were powdered and extracted with 96 % ethanol (3x, each 24 h) by a maceration method at a room temperature. The dilute ethanol extract was evaporated under lower pressure at the temperature of 50 °C to concentrate the extract for toxicity studies.

### 2.4 Phytochemical screening

Phytochemical screening of the *P. feei* root extract was performed to identify secondary metabolites present in the extract following the procedures described by Harbone [9]. The secondary metabolites evaluated were alkaloid, flavonoid, saponin, tannin, quinone, steroid and triterpenoid.

### 2.5 Acute toxicity test

Mice were divided into five groups based on the doses of the *P. feei* root extract (1000, 2000, 4000 and 5000 mg/kg of body weight, five mice per group). The mice in the control group were given a vehicle of 1% Arabic gum suspension. The mortality of the mice was observed, and the organ weight was measured over 14 days. On the day 15th, all animals were anaesthetised and sacrificed, and the vital organs were removed, cleaned with saline and weighed.

### 2.6 Subchronic toxicity test

Subchronic toxicity was evaluated following the guidelines of INADFC [8]. The rats consisted of a control group, three test groups, and two satellite groups. The test groups were administered the extract of the *P. feei* roots orally per day in a 90-day treatment at doses of 100, 400 and 800 mg/kg of body weight, and the control rats received a vehicle of 1% Arabic gum suspension. The satellite groups consisted of a satellite control group and a satellite test group (800 mg/kg, given once per day for 90 days and allowed to alive for 28 days after treatment). On day 91 and 118 (satellite group), all rats were sacrificed, and blood samples were collected for evaluation of biochemical and haematological changes. The organs were dissected immediately, removed, weighed and preserved in 10 % formalin for further histopathology observation. The organ weight was calculated to determine relative organ weights (g/100 g of body weight).

### 2.7 Haematological analysis

Blood samples were analysed to determine red blood cell (RBC) count, white blood cell (WBC) count, haemoglobin (Hb), haematocrite (Hct) and platelet (Plt) according to the guidelines of INADFC [8].

### 2.8 Biochemical analysis

The blood samples were collected in a serum separator tube and centrifuged for 15 min to obtain serum, and the serum was used for determination of creatinine (CREA), urea, SGOT, SGPT, cholesterol (CHO) and triglyceride (TG) according to the INADFC guidelines [8].

### 2.9 Histopathological study

Tissues of the liver, kidney, heart and spleen were washed in normal saline and fixed in 10 % formalin solution for 24 h, then dehydrated in alcohol, embedded in paraffin, and thinly sliced to a thickness of 4–5 μm. The slices were stained with haematoxylin and eosin and observed under the microscope. Histopathological examination was conducted in the Animal Biosystem Laboratory, Departement of Biology, Faculty of Mathematics and Natural Sciences, Padjadjaran University.

### 2.10 Statistical analysis

Data were analysed using one-way analysis of variance (ANOVA) and continued by a post hoc least-significant difference test. The data are presented as mean ± standard error of mean (S.E.M.). A *p*-value of less than 0.05 were considered significantly different.

### 2.11 Ethical approval

The experimental protocols were approved by the Research Ethics Committee of Faculty of Medicine, Universitas Padjadjaran, Indonesia (No. 325/UNG.KEP/EC/2019).

### 2.12 Declaration of compliance

The authors have declared compliance with animal welfare and ethical guidelines.

## 3 Results

### 3.1 Extraction

The extraction of the *P. feei* roots (4500 g) with 96 % ethanol yielded a concentrated extract of 1232 g or a rendement of 27.37 %. The extract was dissolved in 1% of Arabic gum solution just before use for toxicity tests.

### 3.2 Phytochemical screening

Table 1 shows the phytochemicals which belong to the main secondary metabolites present in the extract of *P. feei roots*. Based on colour or precipitation reactions, the compounds identified were flavonoid, saponin, tannin and quinone, polar compounds which may be responsible for the biological activities of the extract, while alkaloid, steroid, and triterpenoid were not detected.

**Table 1:** Secondary metabolites identified in the *P. feei* root extract.

Metabolites	Extract
Alkaloid	–
Flavonoid	+
Saponin	+
Tannin	+
Quinon	+
Steroid	–
Triterpenoid	–

+: Metabolite identified.

-: Metabolite not identified.

### 3.3 Acute toxicity of the *P. feei* root extract

**Fig. 1:**
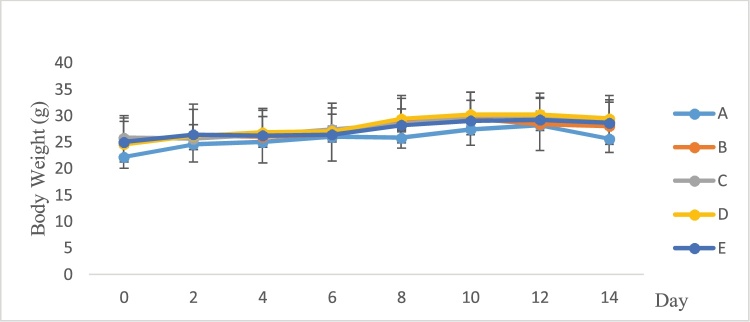
Body weight of mice after treatment with extract. A. Control. B. *P. feei* root extract 1000 mg/kg. C. *P. feei* root extract 2000 mg/kg. D. *P. feei* root extract 4000 mg/kg. E. *P. feei* root extract 5000 mg/kg. All values are mean + SEM (n = 5).

**Fig. 2:**
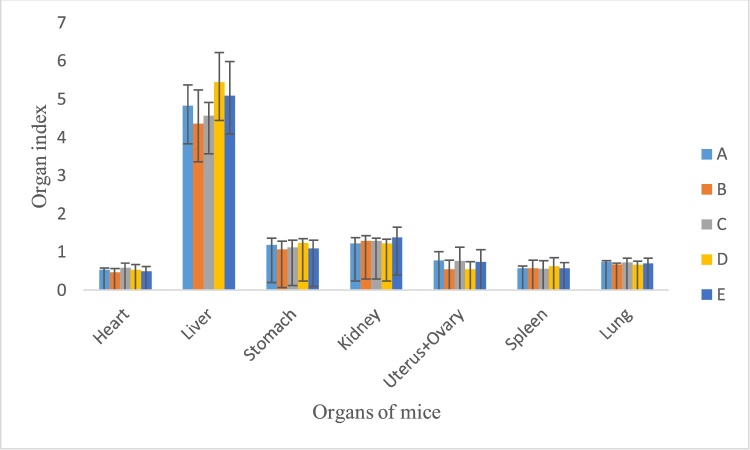
Organ index of mice after treatment with extract. A. Control. B. *P. feei* root extract 1000 mg/kg. C. *P. feei* root extract 2000 mg/kg. D. *P. feei* root extract 4000 mg/kg. E. *P. feei* root extract 5000 mg/kg. All values are mean + SEM (n = 5).

Table 2 indicates none of animals died after single oral doses of the *P. feei* root extract (0% mortality) during the experimental period at all doses (Table 2). Also, there was no evidence of unwanted side effects and toxicity. The body weight (Fig. 1) and internal organ weight (Fig. 2) of the treated mice were not different from the control.

**Table 2:** Acute toxicity of the extract of *P. feei* roots in all groups of mice after 14 days.

Group	Dose (mg/kg)	Mortality of Animals
Control	–	0
EPFR	1000	0
	2000	0
	4000	0
	5000	0

EPFR: Extract of *P. feei* roots (n = 5).

### 3.4 Subchronic toxicity of the *P. feei* root extract

The effect of the *P. feei* root extract on body weight, organ weight, haematological profiles, biochemical parameters and histological features of the organs was observed for 90 days and a further 28 days for the satellite groups.

### 3.5 Effects of the extract of *P. feei* roots on body weight

**Fig. 3:**
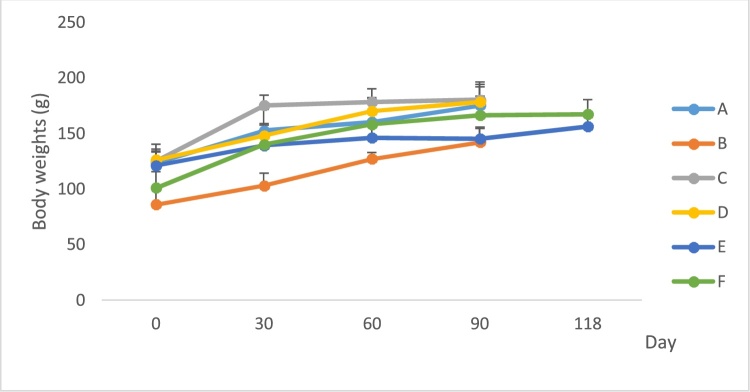
Body weight of female rat after treatment of extract. A. Control. B. *P. feei* root extract 100 mg/kg. C. *P. feei* root extract 400 mg/kg. D. *P. feei* root extract 800 mg/kg. E. Control satellite. F. Satelite 800 mg/kg. All values are mean + SEM (n = 5).

**Fig. 4:**
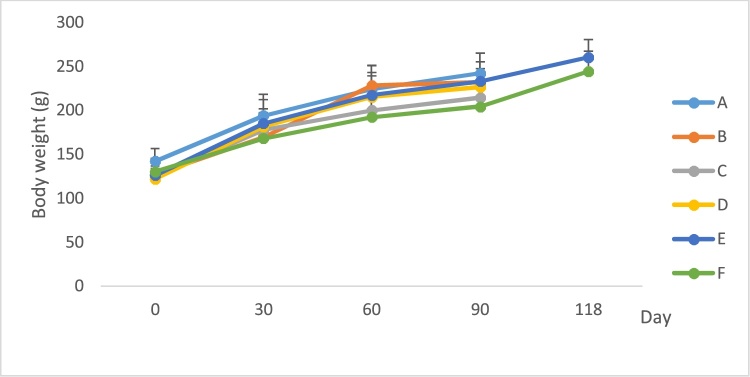
Body weight of male rat after treatment of extract. A. Control. B. *P. feei* root extract 100 mg/kg. C. *P. feei* root extract 400 mg/kg. D. *P. feei* root extract 800 mg/kg. E. Control satellite. F. Satelite 800 mg/kg. All values are mean + SEM (n = 5).

The animals continued to grow normally with the repeated administration of the ethanol extracts of *P. feei roots* for 90 days, with no evidence of detrimental effects on growth (Fig. 3, Fig. 4). This also occurred after the administration of the extract was stopped for 28 days (the satellite groups).

### 3.6 Effects of the *P. feei* root extract on organ weight

**Fig. 5:**
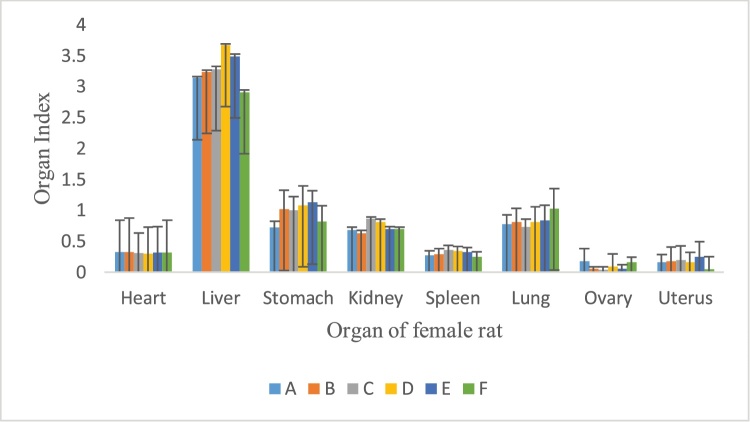
Organ index of female rats after treatment of extract. A. Control. B. *P. feei* root extract 100 mg/kg. C. *P. feei* root extract 400 mg/kg. D. *P. feei* root extract 800 mg/kg. E. Control satellite. F. Satelite 800 mg/kg. All values are mean + SEM (n = 5).

**Fig. 6:**
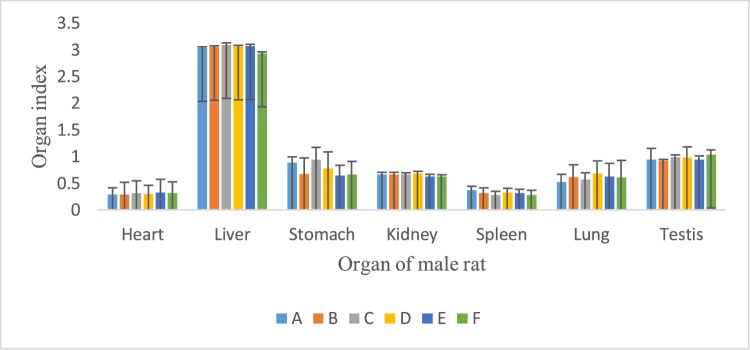
Organ index of male rat after treatment of extract. Control. A. *P. feei* root extract 100 mg/kg. B. *P. feei* root extract 400 mg/kg. C. *P. feei* root extract 800 mg/kg. D. Control satellite. E. Satelite 800 mg/kg. All values are mean + SEM (n = 5).

There was no significant alteration in internal organ weight of the animals as compared with the control following repeated administration of the ethanol extracts of *P. feei roots* at the three doses for 90 days in female and male animals (Fig. 5, Fig. 6).

### 3.7 Effects of the extract of *P. feei* roots on haematological parameters

Haematological parameters of female and male rats are listed in Table 3. In the female and male groups, Hct was higher than the control for the three doses, but this difference was not significant. There was a significant decrease in the Plt count observed in the female group at doses of 400 and 800 mg/kg, but it was not different from the satellite control.

**Table 3:** Haematological parameters in the subchronic toxicity experiment.

Gender	Control	EPFR (mg/kg)			Satelite Control	Satelite EPFR^1^
		100	400	800	–	800
**Female**						
RBC (x10^6^/μL)	8.0 ± 1.19	9.0 ± 1.71	8.4 ± 1.33	7.9 ± 1.14	7.87 ± 1.01	10.7 ± 3.55
Hb (g/dL)	13.0 ± 1.13	13.8 ± 3.3	15.4 ± 0.8	13.2 ± 1.24	12.2 ± 1.21	12.9 ± 0.61
Hct (%)	46.3 ± 3.10	47.1 ± 6.2	48.3 ± 3.1	50.0 ± 2.0	46.2 ± 11.0	44.0 ± 9.10
Plt (x10^5^/μL)	7.88 ± 1.29	7.3 ± 1.50	5.5 ± 1.5^1^	5.8 ± 2.10^1^	6.36 ± 1.17	7.03 ± 1.74
WBC (x10^3^/μL)	10.7 ± 6.28	12.6 ± 5.3	13.6 ± 7.4	10.8 ± 6.73	9.36 ± 4.08	10.70 ± 3.5
**Male**						
RBC (x10^6^/μL)	7.11 ± 1.18	8.6 ± 1.07	8.3 ± 1.02	7.56 ± 1.96	7.44 ± 1.09	7.53 ± 1.03
Hb (g/dL)	14.72 ± 1.2	12.9 ± 2.3	14.2 ± 1.9	14.2 ± 1.04	14.0 ± 1.05	12.8 ± 0.86
Hct (%)	43.0 ± 9.00	49.1 ± 3.0	47.0 ± 8.0	48.0 ± 2.03	47.0 ± 9.13	44.0 ± 10.2
Plt (x10^5^/μL)	5.61 ± 2.72	4.58 ± 1.2	6.47 ± 0.8	5.89 ± 4.08	6.40 ± 1.71	5.81 ± 1.47
WBC (x10^3^/μL)	11.24 ± 7.0	10.9 ± 4.3	11.7 ± 5.9	12.9 ± 10.9	9.18 ± 2.49	11.1 ± 2.98

All values are mean + SEM (n = 5).

EPFR: Extract of *P. feei* roots.

Satelite EPFR: Administration of the extract was continued for 28 days.

1 Significantly different from the control, p < 0.05.

### 3.8 Effects of the *P. feei* root extract on clinical biochemical parameters

The clinical blood chemistry analysis of female and male rats is provided in Table 4, showing no significant alteration in the creatinine, SGOT, SGPT, total cholesterol and triglyceride values in both sexes, but there was a significant increase in the concentration of urea in the female group administered 400 and 800 mg/kg extract. Moreover, the urea concentration in the satellite group treated with 800 mg/kg was significantly increased as compared to that of the satellite control.

**Table 4:** Biochemical parameters in the subchronic toxicity experiment.

Gender	Control	EPFR (mg/kg)			Satelite Control	Satelite EPFR^1^
		100	400	800	–	800
**Female**						
CREA	0.50 ± 0.19	0.60 ± 0.23	0.80 ± 0.16	0.80 ± 0.33	0.60 ± 0.31	0.80 ± 0.19
UREA	19.17 ± 3.59	19.40 ± 3.06	19.80 ± 3.39	22.20 ± 2.19*	19.87 ± 0.13	21.40 ± 2.91*
SGOT	207.6 ± 28.78	232.0 ± 21.89	200.4 ± 59.63	193.6 ± 7.40	234.2 ± 11.07	214.8 ± 16.08
SGPT	62.00 ± 3.53	73.00 ± 13.09	64.00 ± 11.59	63.20 ± 9.36	67.00 ± 4.52	58.80 ± 6.72
CHO	67.21 ± 11.20	70.40 ± 10.64	58.80 ± 8.20	64.20 ± 5.17	60.00 ± 9.38	74.80 ± 13.50
TG	80.40 ± 19.02	89.2 ± 15.83	113.4 ± 46.59	91.00 ± 19.27	98.00 ± 27.19	69.40 ± 13.20
**Male**						
CREA	0.70 ± 0.38	0.80 ± 0.24	0.80 ± 0.16	0.80 ± 0.33	0.70 ± 0.36	0.85 ± 0.14
UREA	22.10 ± 5.30	19.50 ± 3.05	18.30 ± 2.48	19.60 ± 3.00	21.30 ± 4.28	18.90 ± 5.06
SGOT	198.8 ± 22.37	224.2 ± 49.34	217.2 ± 17.04	194.2 ± 20.35	217.0 ± 55.77	213.6 ± 11.39
SGPT	78.40 ± 18.07	74.20 ± 13.70	62.60 ± 9.36	61.20 ± 8.52	78.80 ± 14.41	78.20 ± 15.54
CHO	61.00 ± 10.18	49.80 ± 14.62	60.20 ± 7.73	54.00 ± 5.52	62.00 ± 6.28	68.40 ± 8.38
TG	110.4 ± 43.02	99.00 ± 50.69	117.2 ± 23.85	73.00 ± 1.22	103.6 ± 23.36	104.2 ± 16.44

All values are mean + SEM (n = 5).

EPFR: Extract of *P. feei* roots.

Satelite EPFR: Administration of the extract was continued for 28 days.

1 Dose: mg/kg, bw.

* Significantly different from the control, p < 0.05.

### 3.9 Macropathological observation

Macroscopic examination on the liver and kidney of the animals treated with the *P. feei* root extract did not show abnormalities with regard to colour or texture compared to the control animals. However, lesions were found in the stomachs of the female rats treated with 800 mg/kg in both the nonsatellite and satellite groups, whereas lesions were observed in the male rats regardless of dose, but not in the satellite group (Table 5). The lesions were not serious, as indicated by the number of lesions (about 2–3 points).

**Table 5:** Macropathological conditions of the livers, kidneys, and stomachs.

Gender	Control	EPFR mg/kg)			Satelite Control	Satelite EPFR
	–	100	400	800	–	800
**Female**						
Liver	N	N	N	N	N	N
Kidney	N	N	N	N	N	N
Stomach	N	N	N	L	N	L
**Male**						
Liver	N	N	N	N	N	N
Kidney	N	N	N	N	N	N
Stomach	N	L	L	L	N	N

EPFR: Extract of *P. feei* roots.

Satelite EPFR: Administration of the extract was continued for 28 days.

N: Normal; L: Lesion.

### 3.10 Histopathological observation

**Fig. 7:**
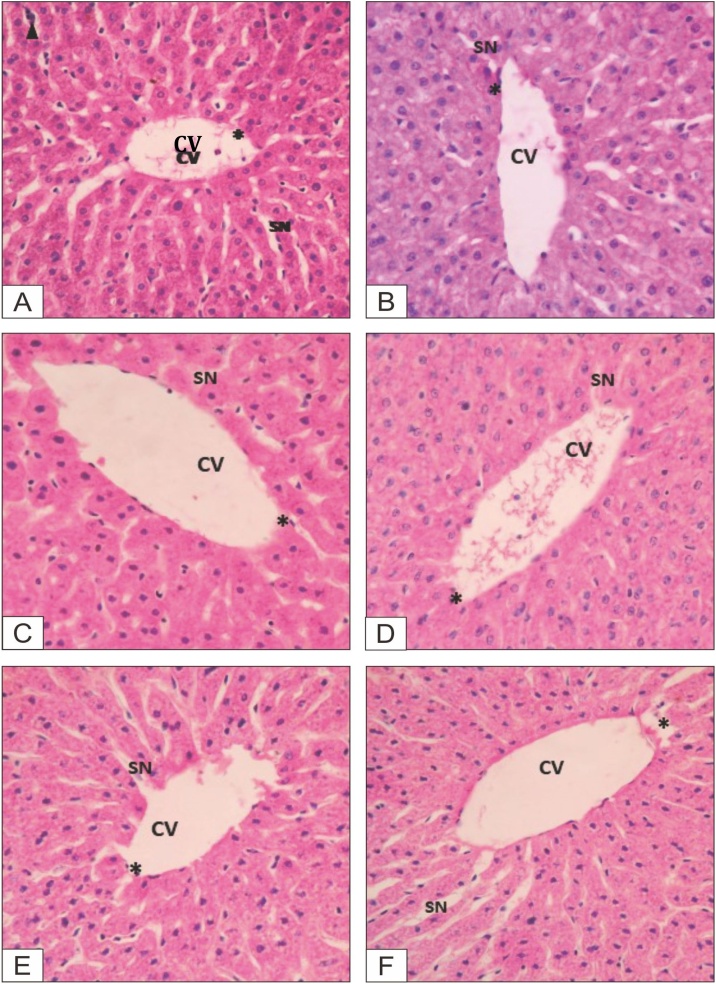
Liver tissues of female animals. **A**. Control group: **B**. Dose 100 mg/kg bw; **C**. Dose 400 mg/kg bw; **D**. Dose 800 mg/kg bw; **E**. Satellite control; **F**. Satellite dose 800 mg/kg bw.

**Fig. 8:**
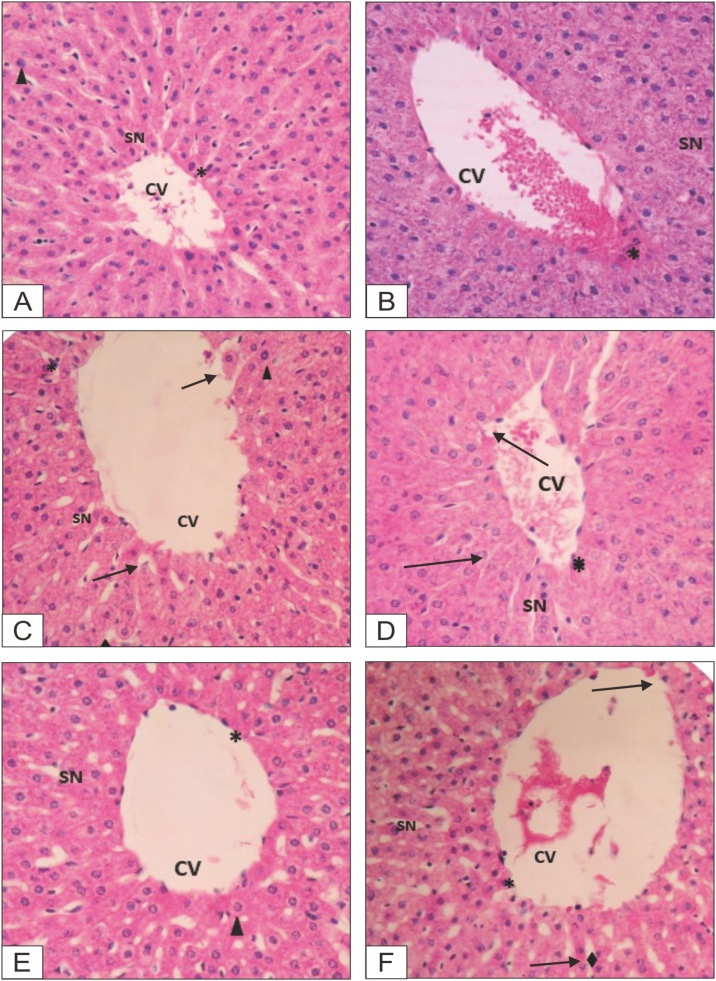
Liver tissues of male animals. **A**. Control group: **B**. Dose 100 mg/kg bw; **C**. Dose 400 mg/kg bw; **D**. Dose 800 mg/kg bw; **E**. Satellite control; **F**. Satellite dose 800 mg/kg bw.

Histopathological observation of the formalin-preserved liver, kidney, heart and spleen samples was conducted, a description of the results is provided in Table 6, with photographs presented in Fig. 7, Fig. 8.

**Table 6:** Description of micropathological data of organ tissues of animals.

Animal group	Sex	Organ	Description
Control	Female	Liver	Vena centralis: normal, sinusoid: normal, and apoptosis: few apoptotic cells
		Kidney	Glomerulus: normal, capsula bowman: normal, and apoptosis: few apoptotic cells
		Heart	Epicardium: normal, myocardium: normal, endiocardium: normal, and apoptosis: few apoptotic cells
		Spleen	Pulpa white and red, and apoptosis: few apoptotic cells
	Male	Liver	Vena centralis: normal, sinusoid: normal, and apoptosis: few apoptotic cells
		Kidney	Glomerulus: normal, capsula bowman: normal, and apoptosis: few apoptotic cells
		Heart	Epicardium: normal, myocardium: normal, endocardium: normal, and apoptosis: few apoptotic cells
		Spleen	Pulpa white and red, and apoptosis: few apoptotic cells
EPFR, 100 mg/kg	Female	Liver	Vena centralis: normal, sinusoid: not clear, apoptosis: few apoptotic cells
		Kidney	Glomerulus: normal, capsula bowman: normal, and apoptosis: few apoptotic cells
		Heart	Epicardium: normal, myocardium: normal, endocardium: normal, and apoptosis: few apoptotic cells
		Spleen	Pulpa white and red, and apoptosis: few apoptotic cells
	Male	Liver	Vena centralis: normal, sinusoid: not clear, apoptosis: few apoptotic cells
		Kidney	Glomerulus: normal, capsula bowman: normal, and apoptosis: few apoptotic cells
		Heart	Epicardium: normal, myocardium: normal, endocardium: normal, and apoptosis: few apoptotic cells
		Spleen	Pulpa white and red, and apoptosis: few apoptotic cells
EPFR, 400 mg/kg	Female	Liver	Vena centralis: normal, sinusoid: not clear, apoptosis: few apoptotic cells
		Kidney	Glomerulus: normal, capsula bowman: normal, and apoptosis: few apoptotic cells
		Heart	Epicardium: normal, myocardium: normal, endocardium: normal, and apoptosis: few apoptotic cells
		Spleen	Pulpa white and red, and apoptosis: few apoptotic cells
	Male	Liver	Vena centralis: minor lesion, sinusoid: not clear, apoptosis: few apoptotic cells
		Kidney	Glomerulus: normal, capsula bowman: normal, and apoptosis: few apoptotic cells
		Heart	Epicardium: normal, myocardium: normal, endocardium: normal, and apoptosis: few apoptotic cells
		Spleen	Pulpa white and red, and apoptosis: few apoptotic cells
EPFR, 800 mg/kg	Female	Liver	Vena centralis: normal, sinusoid: not clear, apoptosis: few apoptotic cells
		Kidney	Glomerulus: normal, capsula bowman: normal, and apoptosis: few apoptotic cells
		Heart	Epicardium: normal, myocardium: normal, endiokardium: normal, apoptosis: few apoptotic cells
		Spleen	Pulpa white and red, and apoptosis: few apoptotic cells
	Male	Liver	Vena centralis: minor lesion, sinusoid: not clear, apoptosis: few apoptotic cells
		Kidney	Glomerulus: normal, capsula bowman: normal, and apoptosis: few apoptotic cells
		Heart	Epicardium: normal, myocardium, endiocardium: normal, and apoptosis: few apoptotic cells
		Spleen	Pulpa white and red, and apoptosis: few apoptotic cells
Satelite Control	Female	Liver	Vena centralis: normal, sinusoid: normal, and apoptosis: few apoptotic cells
		Kidney	Glomerulus: normal, capsula bowman: normal, and apoptosis: few apoptotic cells
		Heart	Epicardium: normal, myocardium: normal, endiocardium: normal, and apoptosis: few apoptotic cells
		Spleen	Pulpa white and red, and apoptosis: few apoptotic cells
	Male	Liver	Vena centralis: normal, sinusoid: normal, and apoptosis: few apoptotic cells
		Kidney	Glomerulus: normal, capsula bowman: normal, and apoptosis: few apoptotic cells
		Heart	Epicardium: normal, myocardium: normal, endiocardium: normal, and apoptosis: few apoptotic cells
		Spleen	Pulpa white and red, and apoptosis: few apoptotic cells
Satelite EPFR, 800 mg/kg	Female	Liver	Vena centralis: normal, sinusoid: normal, and apoptosis: few apoptotic cells
		Kidney	Glomerulus: normal, capsula bowman: normal, and apoptosis: few apoptotic cells
		Heart	Epicardium: normal, myocardium: normal, endiocardium: normal, and apoptosis: few apoptotic cells
		Spleen	Pulpa white and red, and apoptosis: few apoptotic cells
	Male	Liver	Vena centralis: minor lesion, sinusoid: not clear, apoptosis: few apoptotic cells
		Kidney	Glomerulus: normal, capsula bowman: normal, and apoptosis: few apoptotic cells
		Heart	Epicardium: normal, myocardium, endiocardium: normal, and apoptosis: few apoptotic cells
		Spleen	Pulpa white and red, and apoptosis: few apoptotic cells

## 4 Discussion

The evaluation of the toxicity of natural products is an important initial step in screening natural products for pharmacological activity [10]. The toxicity data obtained should increase consumer confidence regarding their safety, in particular, for the use in the development of pharmaceuticals [11]. Typically, the LD_50_ value, which is defined as the dose of chemical agents that kills 50 % of the experimental animals, is used as an indicator of acute toxicity of the agents [12,13]. The determination of the LD_50_ is usually the first step in the toxicity tests conducted for every chemical or medicinal plant before further toxicity tests are carried out [12,14], and the result is used as a guide in the selection of doses for long term toxicity studies and for other studies that use animals [13].

In this study, no mortality of mice occurred at any dose used indicated that the LD_50_ value of the extract was estimated to be higher than 5000 mg/kg, therefore regarded as practically nontoxic. Kennedy et al. (1986) defined toxicity categories of chemicals from extremely toxic to practically nontoxic, and the LD_50_ value greater than 5000 mg/kg belongs to practically nontoxic [15]. In addition, the changes in body weight and organ weight of the extract-treated mice throughout the experimental period were not different from those of the control.

Subchronic tests were performed to assess the undesirable effects of the extracts or compounds administered repeatedly within a certain period of time on experimental animals [10]. The tests provide information regarding target organ toxicity and are designed to identify potentials adverse effects. In this study, after the extract exposure for 90 days, the body weight increment and water or food consumption of animals when compared to the control, indicating that the *P. feei root* extract did not interfere with the metabolism of animals.

Furthermore, no significant change in the internal vital organs (heart, liver, stomach, kidney, spleen, lung, ovary and uterus) weight of animals of both sexes suggested that the *P. feei root* extract at the repeated doses did not give an adverse effect on the normal growth. The extract was not toxic to the mentioned organs because of no significant reduction in the organ weight of the treated animals throughout the extract treatment. These results were confirmed by the histopathological data presented in Table 6.

The blood system has important functions in delivering oxygen to all body tissues, maintaining vascular integrity and providing necessary immune factors for host defence reaction and so on [16]. It is very sensitive to toxic compounds and can be altered by the ingestion of some toxic plants [17]. All parameters measured in this study, counts of red blood cells (RBC), haemoglobin (Hb), haematocrite (Hct), platelet (Plt) and white blood cells (WBC), had values within the normal limits, except, based on the statistical analysis, the platelet count was significantly decreased in the female animals treated with the doses of 400 and 800 mg/kg at the p value of <0.05. However, this effect was reversible as the platelet count returned to normal in the satellite group, suggesting that the *P. feei roots* extract did not cause haematological defects.

It is important to evaluate liver and kidney functions in the assessment of drug toxicity as those organs are vital to the survival of the organism [18]. In this study, clinical biochemistry examinations were conducted to observe alterations in the liver and kidney functions affected by the extract. Parameters measured were creatinine, urea, SGOT, SGPT, cholesterol and triglyceride. In the kidney function examination, the statistical analysis indicated no significant change in the creatinine concentration when compared to the control, but the urea level in the female group was significantly increased by the dose of 800 mg/kg at the *p*-value of <0.05. The higher urea concentration was also found in the satellite group of 800 mg/kg, which was different significantly from that of the satellite control. These data might indicate the occurrence of undesirable effects of the *P. feei roots* extract at the subchronic doses to the kidney. This might be related to the chemical substances of the extract. As mentioned above, the extract of *P. feei roots* contains the proanthocyanidin shellegueain A, which has potential analgesic and anti-inflammatory activity [2]. The analgesic and anti-inflammatory drugs commonly give adverse side effects on the stomach and kidney [19]. However, this study showed that the undesirable effect on the kidney might not be strong as the creatinine concentration remained in the normal limits. This evidence was supported by histopathological observations which showed no alteration in the kidney tissues. However, regarding the rise in the urea levels observed in the female animals, the long-term use of *P. feei* root extract must be careful.

The change in SGOT and SGPT values is a sensitive index to indicate the degree of liver cell damage [20]. The SGOT and SGPT values would be released from the liver cells to the bloodstream when the liver cells get damaged or the chronic liver injury occurred, resulting in the increase in the content of serum [21,22]. In this study, no significant alteration in the SGOT and SGPT values in animals of both sexes indicated that the extract of *P. feei roots* at all doses in the subchronic administration did not influence the hepatocyte function in the animals. The metabolism of fats was not adversely affected by the extract as the cholesterol and triglyceride values remained in normal limits.

In the macropathological observation, lesions were only found in the stomach, and no alteration in the liver and kidney. The lesions in the stomach were assumed due to proanthocyanidin identified as an analgesic and anti-inflammatory agent in the extract of *P. feei* roots. However, this effect was only induced in long-term administration of the extract for 90 days, and in the acute doses given within 60 min, in fact, the extract gives a protective effect on the occurrence of gastric ulcers induced by stress and HCl/ethanol [5]. This gastroprotective effect of the extract in the acute treatment might be due to the unknown compounds of nonspecific chemical substances contained in the extract.

Based on the histopathological observation, there was no pathological abnormalities in the histological tissues of the organs (liver, kidney, heart, and spleen) of the extract treated animals. Only minor lesions were found in the liver tissues, but the lesions did not alter the hepatocytes as the SGOT and SGPT levels remained normal. Further studies could be conducted to evaluate other pharmacological activity on

## 5 Conclusion

This toxicity study revealed that the extract of *P. feei* roots is not toxic in animals and is safe for use as herbal medicine. However, on exposure to the extract for 90 days, the 400 and 800 mg/kg doses significantly increased the urea concentration and caused lesions in the stomachs of female animals, so the extract is recommended for use at doses lower than 400 mg/kg. These results would increase consumer confidence regarding the guarantee of the safety of this plant. In a further study, it is suggested to perform other toxicity tests, such as teratogenic, mutagenic, and reproductive toxicity tests to obtain a broad spectrum term of toxicity profiles of this plant. In addition, a cytotoxicity assay is also possible to be conducted against cancer cell lines [23].

## Declaration of Competing Interest

The authors declare no conflict of interest.
